# Regeneration of periodontal bone defects with mesenchymal stem cells in animal models. Systematic review and meta-analysis

**DOI:** 10.1007/s10266-022-00725-5

**Published:** 2022-07-05

**Authors:** Luis Chauca-Bajaña, Byron Velasquez-Ron, Inmaculada Tomás-Carmona, Fabio Camacho-Alonso, Alba Pérez-Jardón, Mario Pérez‐Sayáns

**Affiliations:** 1 Faculty of Medicine and Dentistry, Oral Medicine, Oral Surgery and Implantology Unit, Periodontics and Implantology Oral Research, College Dentistry, Universidad de Guayaquil, Guayaquil, Ecuador; 2 Dental Prosthesis Department Research, College Dentistry, University of the Americas, UDLA. Av, Colon y 6 de diciembre, Campus Colón, Quito, Ecuador; 3 Oral Sciences Research Group, Department of Surgery and Medical-Surgical Specialties, School of Medicine and Dentistry, University of Santiago de Compostela, Health Research Institute of Santiago (IDIS), Santiago de Compostela, Spain; 4 Department of Oral Surgery, University of Murcia, Murcia, Spain; 5 Oral Medicine, Oral Surgery and Implantology Unit (MedOralRes), Faculty of Medicine and Dentistry, Universidade de Santiago de Compostela, Health Research Institute of Santiago de Compostela (IDIS), (ORALRES GROUP), Santiago de Compostela, A Coruña, Spain

**Keywords:** Stem cells, Pluripotent stem cells, Periodontal defect, Periodontal regeneration

## Abstract

The aim of this study was to evaluate the efficacy of mesenchymal stem cells (MSCs) in the regeneration of periodontal bone defects in animal models. A systematic review and meta-analysis were conducted following the PRISMA guidelines, and the study was recorded in PROSPERO under reference number CDR42021247462. The PICO question was: is periodontal regeneration (cementum, periodontal ligament and alveolar bone) with MSCs more effective than other techniques? Three groups were considered: Group 1: MSCs alone or mixed with regenerative materials. Group 2: only regenerative materials. Group 3: no regenerative material nor MSCs. The search was conducted using MeSH with a total of 18 articles for qualitative analysis and 5 for quantitative analysis. For the meta-analysis, a modification of the effect size algorithm was developed, which considered a comparison of means between treatments using the Student's t sample distribution. When comparing the effect size between Group 1 and Group 2, the effect size for the new cementum was 2.83 mm with an estimated confidence interval of 95% (CI 95%) between 0.48 and 5.17 mm. When considering the fit to a random-effects model, the combined variance (*τ*^2^) was 6.1573 mm, with a standard deviation (SD) of 5.6008 mm and a percentage of total heterogeneity *I*^2^ of 92.33% (*p* < 0.0001). For new bone, the effect size was 0.88 mm, CI 95% − 0.25 to 2.01 mm, *τ*^2^ = 1.3108 mm (SD = 1.2021 mm) and *I*^2^ = 80.46%, *p* = 0.0004). With regard to the new periodontal ligament, it was not possible for the meta-analysis to be performed. MSCs have a greater capacity for tissue regeneration in root cementum than in alveolar bone compared to other regenerative materials.

## Background

Periodontitis is a chronic, multifactorial, inflammatory pathology that results in the destruction of the supporting tissues of the tooth [1]. It is estimated that 50% of the European adult population presents with some form of periodontal disease, and that 750 million people worldwide suffer from severe periodontitis [2]. Periodontal regeneration consists of several methods that aid in the reconstruction or reproduction of a lost or damaged part of the supporting tissues [3–5]. There are many surgical techniques and regenerative materials, which include guided tissue regeneration, growth factors, bone materials, among others, and these are considered as promising solutions for the repair and regeneration of tissues in cases of periodontium, bone defects, atrophic alveolar ridge and furcation defects [6]. Regenerative medicine is a medical discipline that is based on new knowledge of mesenchymal stem cells (MSCs) and their ability to become cells of different tissues [7]. MSCs therapy has demonstrated amazing regenerative capacities in orofacial, neurological, corneal, cardiovascular, hepatic, diabetic, renal, muscular dystrophies, and autoimmune diseases [8]. MSCs are classified into two different types: embryonic and postnatal and adult [9] according to their origin or evolutionary state, and, likewise, they are classified into the following classes: totipotent, pluripotent, and multipotent [10, 11] according to their potential. In this context, MSCs show extensive proliferative potential, multipotency, tropism and immunosuppressive functions, as has been suggested by several in vitro and in vivo studies [12]. In addition to regenerating lost alveolar bone, MSCs can also induce the growth of alveolar cementum and periodontal ligament, which involves the complete regeneration of the periodontal complex [13], a process in which platelets also play a crucial role in haemostasis, immune modulation, and repair mechanisms [14].

MSCs may be isolated from different sources, which include bone marrow, blood from the umbilical cord, adipose tissue, pancreas, liver, skeletal muscle, dermis and the synovial membrane. Alternative sources exist, which include amniotic fluid and Wharton´s jelly from the umbilical cord [15]. Recent studies have indicated that there are no morphological or immunophenotypic differences between the cells obtained from these tissues [16]. In the oral cavity, MSCs can be found in the dental pulp (DPMSCs), dental follicle and gingival connective tissue, as well as other areas [17]. Bianchi et al. studied the bio-morphological reaction of human periodontal ligament fibroblasts to different types of dentinal derivates (mineralized dentine, deproteinized and demineralized dentine, and demineralized dentine), and a positive response was observed in terms of proliferation and adhesion, with stronger vinculin and integrin signal. This therefore confirms that dentinal derivates present high conductivity and inductivity properties in the regenerative processes [18].

The use of advanced therapies based on MSCs in periodontal regeneration is derived from pre-clinical investigations, as very few controlled clinical trials (CCT) have been conducted to date to evaluate their efficacy in the treatment of human periodontal lesions [19]. In recent years, a wide variety of studies have been conducted in which MSCs were used in combination with other biomaterials to obtain optimal periodontal regeneration [20–23]; however, none of these achieved optimal success, and conflicting results were reported [24–27].

To the best of the authors’ knowledge, no meta-analysis of preclinical studies on the efficacy of MSCs in periodontal regeneration has been performed to date, and therefore there is a knowledge gap that must be closed in order to lay the foundations for adequate clinical studies in the future.

## Methods

### Protocol and registration

A specific study protocol was designed for the search and data retrieval process, which fulfilled PRISMA guidelines [28]. The protocol was registered in PROSPERO under reference ID CDR42021247462 to minimize the risk of bias and improve the transparency, precision, and integrity.

### Focused question

The review was designed to answer this PICO question: Is periodontal regeneration with MSCs more effective than other techniques? P: Articles with studies of periodontal defects in animals were evaluated; I: Intervention, periodontal regenerations performed with different MSCs, alone or in combination with other biomaterials; C: Comparison of the different results of regeneration of the support periodontal tissue with different regenerative materials; O: Observation, the amount of periodontal regeneration, histologically measured as new bone, cementum and periodontal ligament in the periodontal defect were compared.

### Information sources and search strategy

The search was conducted using the Rayyan QCRI programme (Qatar Computing Research Institute (Data Analytics), Doha, Qatarcon). Following the PRISMA requirements, the MeSH terms used were: “Mesenchymal Stem Cells”, “Periodontal Attachment Loss”, “Periodontal Atrophy”, “Alveolar Bone Loss” and “Guided Tissue Regeneration, Periodontal”. For verification purposes, other keywords (pluripotent stem cells, adult stem cells, hematopoietic stem cells, bone marrow stem cells (BMSCs), mesenchymal stem cell transplantation, furcation defect, bone regeneration) were also included when searching MEDLINE through PubMed, EMBASE through OVID, the Web of Science, Scopus, Cochrane Library, Clinical Trials, the five WHO regional bibliographic databases (AIM, LILACS, IMEMR, IMSEAR, WPRIM), and the Conference Proceedings Citation Index. Any potentially relevant articles that any of the authors were aware of, as well as reference lists from the retrieved articles, were also comprehensively checked. This process was complemented by a manual search (peer-reviewed journals with related content).

### Eligibility criteria

All of the references identified from computerized databases were manually retrieved, and the articles were included if they met the following inclusion criteria: (1) Studies on bone defect regeneration with MSCs (type/origin) in animals without systemic conditions or genetic modification. (2) Studies in dogs, rabbit, rats, and pigs (the gender and age of the animals were not considered in the studies). (3) Studies of preclinical controlled animal models, in which MSCs were used locally to correct periodontal defects in the first six months with a single evaluation at 2—4—6—8 weeks. (4) Data on periodontal regeneration (cementum, bone and periodontal ligament). (5) Studies published in the English language. The following exclusion criteria were considered: (1) Human studies. (2) Alveolar bone regeneration only with biomaterials. (3) Studies that did not include a control group, or studies in which it was not possible to compare the regeneration results due to the absence of data regarding gain/loss of periodontal regeneration. (4) Clinical cases. (5) Studies for which there was a lack of measurements and standard deviation data. (6) Reviews, systematic reviews and meta-analysis.

### Study selection and data extraction process

Data was retrieved by two researchers (LC and MPS) using a custom-made extraction sheet. Any discrepancies that arose between the two researchers were resolved by a third researcher (BVR) who was blinded to the study hypothesis. The following data was recorded: first author, country, type of study, type of animal, number of animals, overall number of defects, type of control condition (including group with other types of regenerative materials), number of defects in the control group, type of MSCs, number of defects in the MSCs group, and periodontal regeneration assessment procedure.

First the title and abstracts of all potential records were read, and a full-text protocol was used to determine the inclusion of any texts with insufficient data. Subsequently, all eligible articles were examined in full text, and if any data considered essential for the review was missing or unclear, an attempt was made to contact the corresponding author of the study in order to resolve or clarify the problem.

### Evaluation of quality and risk of bias

The risk of bias was assessed according to the Systematic Review Centre for Laboratory Animal Experimentation (SYRCLE) [29]. An overall bias risk assessment was conducted for each study included, assigning the following bias ratings: High, Unclear, and Low. The following elements were evaluated for bias: selection, performance, detection, desertion, notification, among others. These elements were assessed using the Cochrane RoB tool. SYRCLE's risk of bias tool for animal studies includes the following aspects: (1) Sequence generation: this was evaluated taking into account whether or not periodontal defects were induced. (2) Allocation concealment: randomization results were checked by evaluating the baseline characteristics in test and control groups. (3) Incomplete result data: the inclusion of all data was verified, including the types of animals (beagle dogs, rats, mini pigs, rabbits), the number of animals, the types of periodontal defects (periodontal bone defects, furcation defects type II and III), types of MSCs, control group or groups with other materials, and assessment of periodontal regeneration (periodontal ligament, root cementum and alveolar bone). (4) Selective reporting of results: the study protocols and group results were evaluated with other materials and other sources of bias [Table 1]. (5) Selective reporting of results: The MSC groups were compared with their respective control groups, to determine whether or not there was an increased number of animals in the MSC groups or the regenerative materials group, whether or not the animals had been given additional medications, the number of surgical interventions performed, and whether or not the animals in each study received the same treatment and care, or if differentiations were made taking into account the different types of animal (beagle dogs, mini pigs, rats and rabbits) and the types of periodontal defects.

**Table 1:**
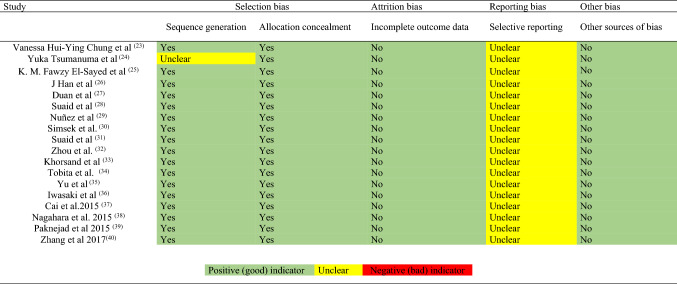
SYRCLE’s Rob tool for each experimental animal studies (*n* = 18)

### Statistical analysis

#### Qualitative analysis

The qualitative analysis described the general aspects of the articles that met the inclusion criteria. A systematic review of the included articles was carried out, describing the previously defined characteristics (see data extraction), and distinguishing between the three study groups: (1) the first group of MSCs alone or mixed with other types of regenerative materials. (2) the second group of other regenerative materials. (3) the third group in which no regenerative material was placed. The periodontal regeneration (alveolar bone, periodontal ligament, and alveolar cementum) was assessed in each group.

#### Meta-analysis

Meta-analysis was used under the following systematic process: (1) Definition of experimental variables. (2) Identification of treatment and control. (3) Determining the effect size and its standard deviation. (4) Specification of the model according to the type of characteristics: qualitative or quantitative. (5) Selection of the fixed or random effects model. 6) Model validation and heterogeneity analysis. (7) Graphical representations (Forest Plot and Funnel Plot). (8) Interpretation of the results. This process was validated through the following assumptions: (1) Tissue regeneration variable measured in mm and (2) Modification of the effect size algorithm, considering a comparison of means between treatments with Student's *t* sample distribution.

With respect to the method used for the meta-analysis, the effect size was determined for each of the five included studies. A high variability was obtained given that in the process of calculating the effect size, the control was not used, and in contrast, comparison between Groups 1 and 2 was made. This methodological change was justified due to the extent to which the articles reviewed did not contain adequate analysis of the control treatment, and due to the lack of extensive literature available in order to guarantee the probabilistic properties of the sampling distribution of the statistic effect size. This was adjusted for both the New Cementum and the New Bone. The calculation process was carried out through the R software, using the following statistical packages: "meta" and "metasens" [30].

## Results

### Biographical research

**Fig. 1:**
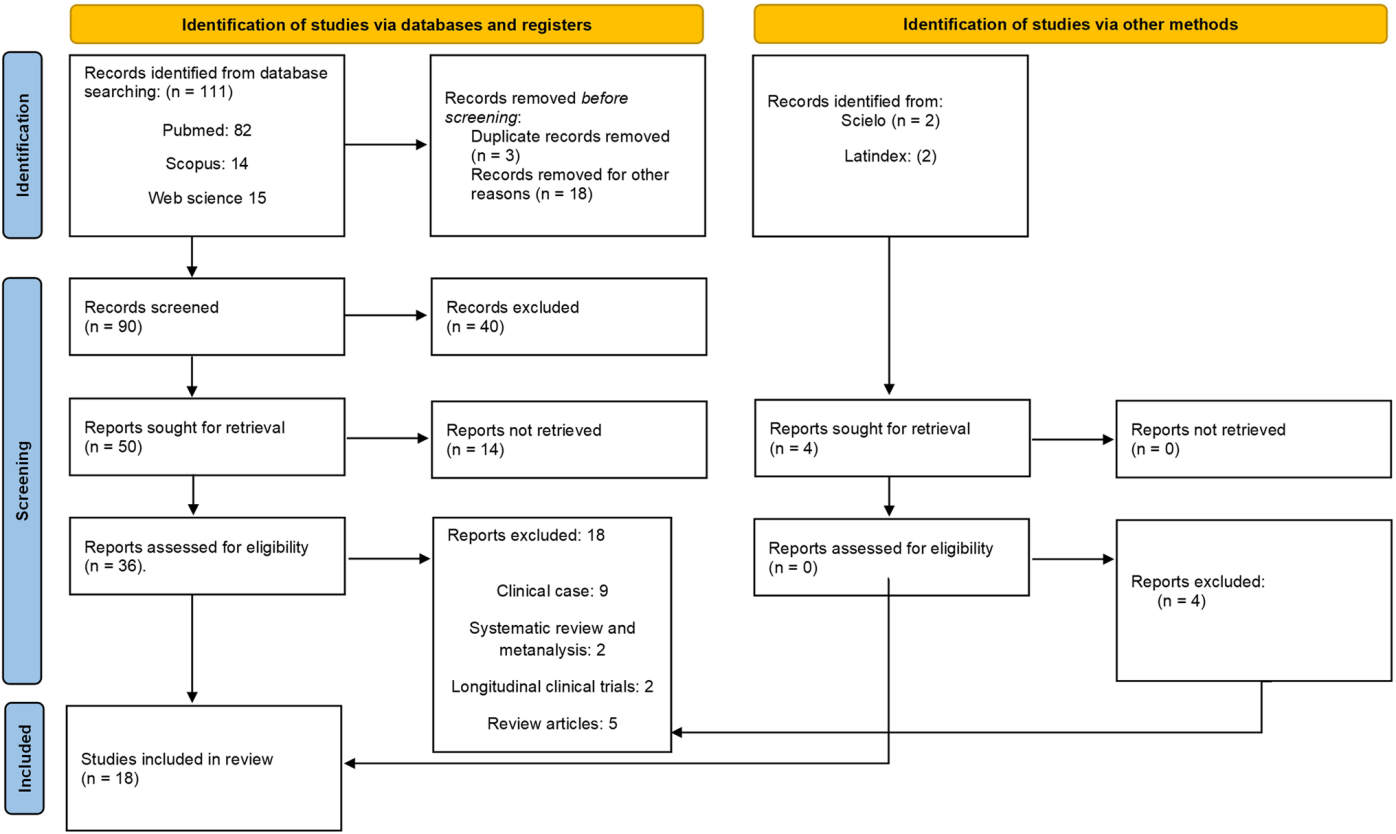
Flowchart of selected studies

111 articles were identified through the aforementioned search, the abstracts of which were reviewed for content relevant to the topic under study, with 93 of the articles excluded for this reason. After the critical analysis of the studies had been performed, 18 studies from different geographical areas met the inclusion criteria. These 18 articles were included for qualitative analysis, and 5 of said articles were included for meta-analysis (Fig. 1).

### Risk of bias

The articles were evaluated using the SYRCLE RoB tool for animal studies [29]. It was determined that all of the studies had a low risk of bias [31–48], in terms of sequence generation, allocation concealment, incomplete outcome data and their sources of bias. The complete data can be found in Table 1.

### Qualitative analysis: Clinical features and periodontal regeneration

The first group of MSCs alone or mixed with other types of regenerative materials included several materials, such as polyglycolic acid, bone bovine, gelatin sponge, apatite-coated silk, autogenous cortical bone, platelet-rich plasma, polyglycolic acid/trimethylene carbonate, beta-tricalcium phosphate, hydroxyapatite, and biomimetic intrafibrillarly mineralized collagen. The MSCs were obtained from the dental pulp, periodontal ligament, gingival margin and bone marrow, periosteal alveolar cells. The second group of other regenerative materials was comprised of several products: bovine bone, adenovirus, beta-tricalcium phosphate, platelet rich plasma (PRP), polyglycolic acid/trimethylene carbonate, polyglycolic acid and osteoprotegerin. Periodontal regeneration was based on the results obtained by the gain of alveolar bone, periodontal ligament and alveolar cementum tissue and the results are included in full in Table 2.

**Table 2:** Full data of the studies included in the systematic review

Author (year)	Country	Type of study	Animal model	No of global defects	Type of control condition	Defects in controls	Type of target condition MSCs	Defects with stem cell	Type of target condition other techniques	Defects with other techniques	Periodontal regeneration
Chung et al. 2011 [23]	Taiwan	Case–control A split mouth	Beagle dogs	18 mandibular 4 mm defects	No treatment	No treatment	AdvBMP-2 group with BMP-2 expressing MSC Other group MSC alone	9 bilateral mandibular defects of 4 mm And other group 9 defects	No treatment	No treatment	Bone new MSC alone: 578.69 ± 68.13 advBMP-2 group with BMP-2 expressing MSC: 282.73 ± 36.68 mm3
Tsumanuma et al. [24]	Japan	Case–control A split mouth	Beagle dogs	1-wall intrabony defects (5 5 mm in depth, mesio-distal width) were created surgically on the mesial and distal sides of mandibular third premolars and the mesial of mandibular first molars bilaterally	No treatment	No treatment	PDLC + β-TCP / collagen other group BMMSC + β-TCP / collagen other group (APC) + β-TCP / collagen	1st group 1 defects 2nd group 1 defects 3rd group 1 defects	Pga	1 defects	Newly formed cementum thickness (mm) PDLC + β-TCP / collagen: 14.37 ± 4.38 mm BMMSC: 7.80 ± 2.67 mm APC + β-TCP/collagen:3.99 ± 2.64 mm Pga: 6.59 ± 2.82 mm Periodontal score (1–5) PDLC + β-TCP/collagen: 4.00 ± 1.41 BMMSC: 3.38 ± 0.95 APC + β-TCP/collagen: 2.63 ± 0.48 Pga: 1.75 ± 0.96 Bone regeneration ratio (%) PDLC + β-TCP/collagen: 72.28 ± 32.56 BMMSC: 2.05 ± 12.92 APC + β-TCP/collagen: 67.63 ± 21.56 Pga: 67.51 ± 14.26 Length of junctional epithelium (mm) PDLC + β-TCP/collagen: 0.15 ± 0.30 (BMMSC: 0.49 ± 0.97 APC + β-TCP/collagen: 0.63 ± 0.52 Pga: 0.64 ± 0.46
Fawzy El-Sayed et al. [25]	Germany	Case–control A split mouth	Mini pings	48 Bilateral fenestration defect	SRP + surgical access only	8 defects Other group 8 defects	SRP + ABBM + GM-MSC + collagen membrane Other group SRP + collagen + GM-MSC + collagen membrane	8 defects and other group 8 defects	SRP + ABBM + collagen membrane Other group SRP + collagen membrane	8 defects and other group 8 defects	Periodontal regeneration Negative control: − 5.4 ± 1.9 mm Surgical access: − 4.9 ± 2.1 mm ABBM + membrane: − 4.7 ± 2.1 mm Collagen membrane: − 6.2 ± 2.0 mm ABBM + GM-MSC + membrane: − 1.6 ± 1.1 mm Collagen + GM-MSC + membrane: − 2.4 ± 1.7 mm
J Han et al. [26]	Australia	Case–control A split mouth	Rats	72 defects Fenestration defects were surgically created on right first and second mandibular molars (W × L = 2 × 3 mm)	No treatment	24 defects	Gelatin sponge + PDL-MSC	24 defects	Gelatin sponge	24 defects	% of bone fill − 7 days Untreated: 0% Gelatin sponge: 0% Gelatin sponge + PDL-MSC: ~ 5% − 14 days Untreated: ~ 10% Gelatin sponge: ~ 20% Gelatin sponge + PDL-MSC: ~ 50% − 21 days Untreated: ~ 20% Gelatin sponge: ~ 30% Gelatin sponge + PD-MSC: 60% % of new bone length in the defect − 7 days Untreated: ~ 15% Gelatin sponge: 0% Gelatin sponge + PDL-MSC: ~ 15% − 14 days Untreated: ~ 25% Gelatin sponge: ~ 30% Gelatin sponge + PDL-MSC: ~ 75% − 21 days Untreated: ~ 30% Gelatin sponge: ~ 40% Gelatin sponge + PDL-MSC: 80% New cementum (mm) − 7 days Untreated: 0.00 mm Gelatin sponge: 0.00 mm Gelatin sponge + PDL-MSC: 0.00 mm − 14 days Untreated: 0.01 mm Gelatin sponge: 0.01 mm Gelatin sponge + PDL-MSC: 0.01 mm − 21 days Untreated:0.05 mm Gelatin sponge: 0.1 mm Gelatin sponge + PDL-MSC: 0.3 mm
Duan et al. [27]	China	Case–control A split mouth	Rats	36 defects	Group control with apatite-coated silk	6 defects	Apatite-coated silk + EMD + iPSC	6 defects Bilateral fenestration defects were surgically created on mandibular first molars (2 × 1.5 mm2)	Apatite-coated silk + EMD	6 defects bilateral	New bone Apatite-coated silk: 39.57 ± 1.58% Apatite-coated silk + EMD: 41.25 ± 2.14% Apatite-coated silk + EMD + iPSC: 58.53 ± 2.6%
Suaid et al. [28]	Brasil	Case control	Beagle dogs	14 defects Bilateral class II furcation defects	No treatment	No treatment	Collagen composite + PDLMSC + resorbable glycode and lactide copolymer membrane	7 defects Bilateral class II furcation defects	Collagen composite + resorbable glycode and lactide copolymer membrane	7 defects Bilateral class II furcation defects	New bone Collagen + membrane: 7.01 ± 0.61mm2 Collagen composite + PDL-MSC + membrane: 9.02 ± 2.30 mm New cementum Collagen membrane: 6.00 ± 1.50 mm Collagen + PDL-MSC + membrane: 8.08 ± 1.08 mm
Nuñez et al. [29]	Spain	Case–control A split mouth	Beagle dogs	24 defects Bilateral 3-wall intrabony defects	No treatment	No treatment	SRP + collagen sponge + CDC Other group SRP + collagen sponge + PDL-MSC	8 defects And other group 8 defects	SRP + collagen sponge	8 defects	New bone collagen sponge: 2.63 ± 0.67 mm collagen sponge CDC: 2.63 ± 0.44 mm collagen sponge + PDL-MSC: 3.08 ± 1.06 mm New cementum collagen sponge: 1.56 ± 0.39 mm collagen sponge + CDC: 3.98 ± 0.59 mm collagen sponge + PDL-MSC: 4.07 ± 0.97 mm
Simsek et al. [30]	Istanbul, Turkey	Case–control A split mouth	Beagle dogs	30 defects Bilateral class II furcation defects	Grouop control SRP	6 defects	SRP + PRP + BM- MSC	6 defects	SRP + autogenous cortical bone Other group SRP + PRP Other group SRP + PRP + autogenous cortical bone	6 defects and other group 6 defects Other group 6 defects	New alveolar bone SRP: 31.98 ± 6.67% SRP + PRP: 33.95 ± 15.39% SRP + autogenous cortical bone: 84.60 ± 4.85% SRP + PRP + autogenous cortical bone: 68.80 ± 14.20% SRP + PRP + BM-MSC: 80.47 ± 8.23% new cementum SRP: 3.33 ± 3.33% SRP + PRP: 36.60 ± 20.1% SRP + autogenous cortical bone: 93.62 ± 4.09% SRP + PRP + autogenous cortical bone: 66.83 ± 10.78% SRP + PRP + BM-MSC: 70.47 ± 11.75%
Suaid et al. [31]	Brasil	Case–control split mouth	Beagle dogs	28 defects Bilateral supracrestal class III furcation defect	Surgical access only	7 defects	Collagen composite + PGA: TMC fiber-PLGA membrane + PDL-MSC	7 defects	PGA: TMC fiber-PLGA membrane Other group: collagen composite + PGA:TMC fiber-PLGA membrane	7 defects and other group 7 defects	New bone Surgical access: 1.89 ± 0.95 mm2 Membrane: 2.91 ± 0.56 mm2 Membrane + collagen: 3.94 ± 1.52 mm2 Membrane + collagen + PDL-MSC: 5.45 ± 1.58 mm2 New cementum Surgical access: 1.70 ± 0.60 mm Membrane: 2.87 ± 0.74 mm Membrane + collagen: 3.66 ± 0.95 mm2 Membrane + collagen + PDL-MSC: 4.82 ± 0.61 mm Periodontal regeneration Surgical access: 0.69 ± 0.59 mm Membrane: 1.52 ± 0.39 mm Membrane + collagen: 2.33 ± 0.95 mm Membrane + collagen + PDL-MSC: 3.43 ± 1.44 mm
Zhou et al. [32]	China	Case–control split mouth	Beagle dogs	24 Bilateral fenestration defects were surgically created at the buccal aspect of mandibular P2-P4. (W × L × D = 4 × 4 × 3 mm)	SRP	6 defects	PLGA + BMMSC + collagen membrane (n = 6) Group 4: PLGA + OPG BM-MSC + collagen membrane (n = 6)	6 defects and other group 6 defects	PLGA + collagen membrane	6 defects	New bone SRP: 0.33 ± 0.09 mm PLGA + membrane: 0.67 ± 0.14 mm PLGA + BM-MSC + membrane: 1.12 ± 0.12 mm PLGA + OPG- BM-MSC + membrane: 2.02 ± 0.11 mm New cementum SRP: 0.44 ± 0.04 mm PLGA + membrane: 0.11 ± 0.11 mm PLGA + BM-MSC + membrane: 1.02 ± 0.05 mm PLGA + OPG-BM-MSC + membrane: 2.02 ± 0.10 mm New connective tissue SRP: 2.12 ± 0.05 mm PLGA + membrane: 2.73 ± 0.09 mm PLGA + BM-MSC + membrane: 2.12 ± 0.11 mm PLGA + OPG-BM-MSC + membrane: 3.34 ± 0.14 mm
Khorsand et al. [33]	Iran	Case–control split mouth	Beagle dogs	20 Bilateral 3-wall defects were surgically created at the mesial aspect of mandibular	No treatment	No treatment	SRP + ABBM + DPMSC	10 DEFECTS	SRP + BONE BOVINE	10 DEFECTS	New bone SRP + ABBM: 3.10 ± 0.82 mm SRP + ABBM + DP-MSC: 3.60 ± 1.06 mm New cementum (mm): SRP + ABBM: 2.42 ± 1.40 mm SRP + ABBM + DP-MSC: 3.83 ± 1.32 mm New PDL SRP + ABBM: 1.77 ± 1.27 mm SRP + ABBM + DP-MSC: 3.30 ± 1.12 mm
Tobita et al. [34]	Japan	Case–control A split mouth	Beagle dogs	48 Bilateral class III furcation defects were surgically created at mandibular P2, P3 and P4	No treatment	4 weeks 8 defects 8 weeks 8 defects	PRP + A-MSC	4 weeks 8 defects 8 weeks 8 defects	PRP	4 weeks 8 defects 8 weeks 8 defects	% of new bone − 4 weeks Untreated: 37.0% PRP: 33.6% PRP + A-MSC: 35.1% − 8 weeks Untreated: 40.3% PRP: 53.7% PRP + A-MSC: 63.9% % of new cementum − 4 weeks Untreated: 38.7% PRP: 37.7% PRP + A-MSC: 36.4% − 8 weeks Untreated: 61.7% PRP: 62.5% PRP + A-MSC: 84.7%
Yu et al. [35]	China	Case–control A split mouth	Beagle dogs	16 defects Bilateral surgically created class III furcation defects at mandibular	SRP	8 defects	SRP + G-MSC sheet	8 defects	No treatment	No treatment	Area of new bone SRP: 10.37 ± 9.53% SRP + G-MSC sheet: 47.11 ± 7.91% % of new cementum length SRP: 24% SRP + G-MSC sheet: 68%
Iwasaki et al. [36]	Japan	Case–control A split mouth	Rats	12 Bilateral class II furcation defects	No treatment	No treatment	PDL-MSC + amniotic membrane	6 defects	amniotic membrane	6 defects	Histological new cementum thickness Amniotic membrane: 0.00 ± 0.00 μm PDL-MSC + amniotic membrane: 3.44 ± 0.49 μm
Cai et al. [37]	China	Case–control A split mouth	Rats	24 Bilateral surgically created 3-wall intrabony defects at the mesial aspect of the maxillary first molars	No treatment	No treatment	undifferenti- ated BM-MSC + PLGA/ PCL Other group osteogenic differentiated BM-MSC (oBM-MSC) + PLGA/PCL Other group chondrogenic differentiated BM-MSC (cBM-MSC) + PLGA/PCL	6 defects, other group 6 defects and other group with stem cells 6 defects	PLGA/PCL	6 defects	Relative new bone area: PLGA/PCL: 0.1 BM-MSC + PLGA/PCL: 0.14 oBM-MSC + PLGA/PCL: 0.22 cBM-MSC + PLGA/PCL: 0.2 Relative functional ligament length: PLGA/PCL: 0.23 BM-MSC + PLGA/PCL: 0.09 oBM-MSC + PLGA/PCL: 0.23 cBM-MSC + PLGA/PCL: 0.16
Nagahara et al. [38]	Japan	Case–control A split mouth	Beagle dogs	72 Bilateral class III furcation defects were surgically created at mandibula	No treatment	No treatment	SRP + BM- MSC + collagen Other group with stem cells: SRP + BM- MSC + β-TCP/collagen	20 defects and 20 defects other group	SRP + collagen Other group SRP + β-TCP/ collagen	10 defects and other group 16 defects	% area of new bone − 4 weeks: Collagen: 17.5 ± 8.3% β-TCP/collagen: 38.5 ± 15.1% BM-MSC + collagen: 23.5 ± 17.4% BM-MSC + β-TCP/collagen: 65.3 ± 13.1% − 8 weeks Collagen: 31.1 ± 4.3% β-TCP/collagen: 49.8 ± 1.4% BM-MSC + collagen: 65.6 ± 19.9% BM-MSC + β-TCP/collagen: 76.6 ± 10.3% % of new cementum − 4 weeks Collagen: 36.8 ± 7.1% β-TCP/collagen: 38.5 ± 12.7% BM-MSC + collagen: 69.9 ± 30.3% BM-MSC + β-TCP/collagen: 79.4 ± 15.7% − 8 weeks Collagen: 56.9 ± 16.3% β-TCP/collagen: 45.4 ± 19.0% BM-MSC + collagen: 89.1 ± 15.1% BM-MSC + β-TCP/collagen: 89.2 ± 10.3%
Paknejad et al. [39]	Mexico	Case–control A split mouth	Beagle dogs	16 Bilateral 3-wall intrabony defects	No treatment	No treatment	SRP + ABBM + BM-MSC	8 defects	SRP + bone bovine	8 defects	New cementum ABBM: 3.33 ± 0.94 mm ABBM + BM-MSC: 2.03 ± 1.30 mm % of cementum length in the defect ABBM: 80.1% ABBM + BM-MSC: 48.5% New PDL ABBM: 2.69 ± 0.73 mm ABBM + BM-MSC: 1.53 ± 1.21 mm % of PDL length in the defect ABBM: 64.3% ABBM + BM-MSC: 36.5% New bone ABBM: 2.70 ± 0.86 mm ABBM + BM-MSC: 1.99 ± 1.3 mm % of bone length in the defect ABBM: 64.7% ABBM + BM-MSC: 48.5%
Zhang, C et al. [40]	China	Case–control A split mouth	Mini pigs	18 defects and 2 defects cranial bone	S/N	6 defects	IMC con PDL-MSC and other group HA con PDL-MSC	6 defects and other group 6 defects	No treatment	No treatment	New bone IMC con PDL-MSC 45,2 +—17,7% HA con PDL-MSC 29,3 +—7,7% Control 19,6 +—3,4%

*ABBM* anorganic bovine bone mineral, *PDLMSC* periodontal ligament stem cells, *SRP* scaling and root planning, *DPSC* dental pulp mesenchymal stem cells, *MSC* mesenchymal stem cells, *PRP* platelet-rich plasma, *BM-MSC* mesenchymal stem cells of the bone marrow, *PGA* polyglycolic acid, *PGA-TMC* polyglycolic acid/trimethylene carbonate, *PLGA* polylactic-co-glycolic acid), *OPG* osteoprotegerin, *A-MSC* adipose tissue-derived, *MSC G-MSC* gingival margin-derived, *β-TCP* beta-tricalcium phosphate, *HA* hydroxyapatite, *IMC* biomimetic intrafibrillarly mineralized collagen

In the qualitative analysis, 18 articles were considered, some of which contained results expressed in percentages, millimetres and square millimetres. 67% of the studies were performed on Beagle dogs, 22% on rats and 11% on mini pigs. In the study groups, the most relevant stem cells were those obtained from the periodontal ligament (PDLMSC) and bone marrow stem cells (BM-MSC) mixed with bovine bone or platelet-rich plasma. In terms of bone defects, the most widely used stem cell type was PDLMSC with bovine bone and in furcation defects, type II and III, BM-MSC with platelet-rich plasma were used. A full summary of the results of the included studies is included in Table 2.

### Meta-analysis

#### New cementum

The results obtained regarding the regeneration of root cementum when comparing the effect size between Group 1–Group 2, with Group 1 being stem cells and Group 2 other regenerative materials, shows that Group 1 presented greater regeneration of periodontal tissue than Group 2. Indeed, the initial results showed that among the studies reviewed, the effect size had an average of 3.4005 mm of tissue with a standard deviation of 1.2634. This means that the average effect size explicitly expresses the highest value by Group 1 compared to Group 2 with characteristics of experimental homogeneity in each of the experiments due to the coefficient of variation not greater than one, | cv |< 1.

**Fig. 2:**
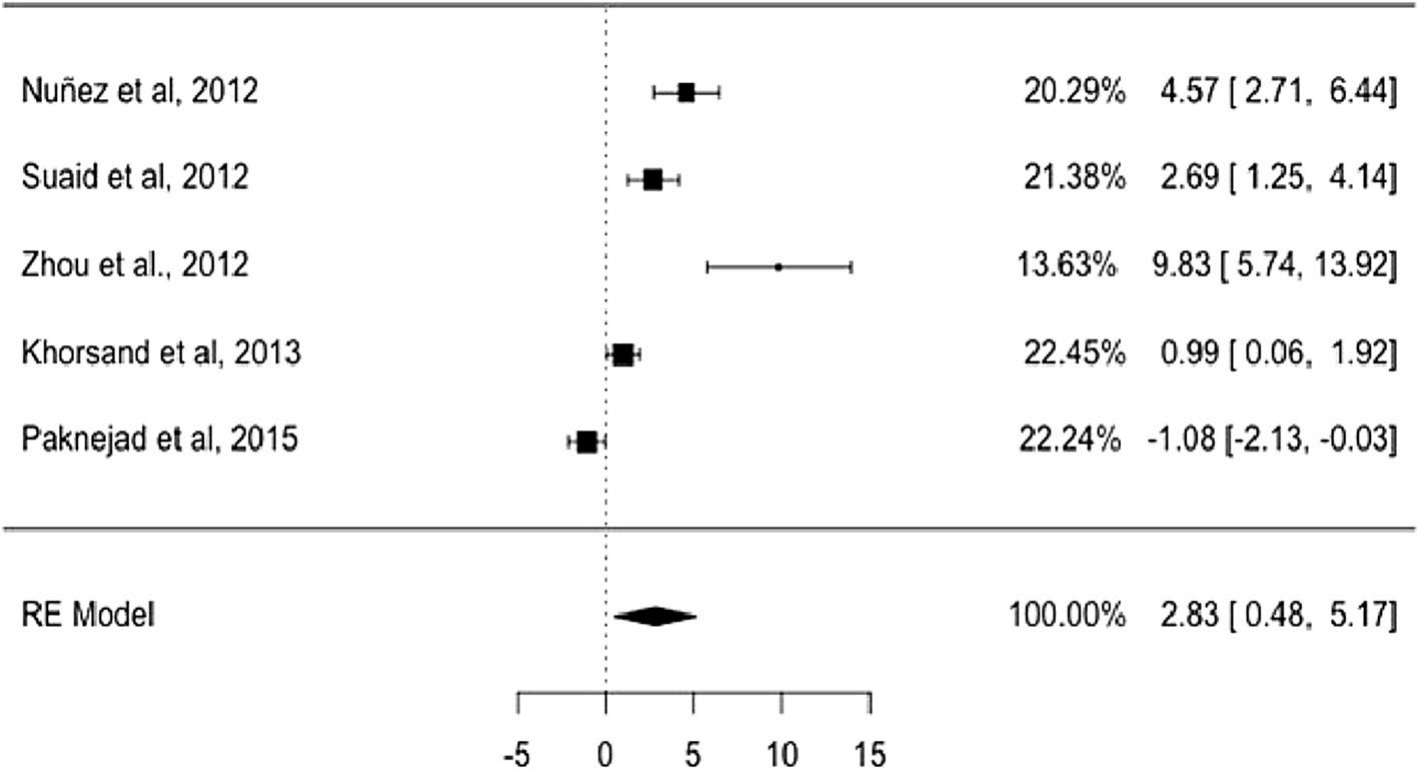
Forest plot and data from the meta-analysis for the regeneration in new cementum. ${\tau }^{2}$ (estimated amount of total heterogeneity): 6.1573 (SE = 5.6008). $\tau$ (square root of estimated ${\tau }^{2}$ value): 2.4814. ${I}^{2}$ (total heterogeneity / total variability): 92.33%. ${H}^{2}$ (total variability/sampling variability): 13.03. Test for Heterogeneity: *Q* (df = 4) = 52.1251, *p*-value < 0.0001

**Fig. 3:**
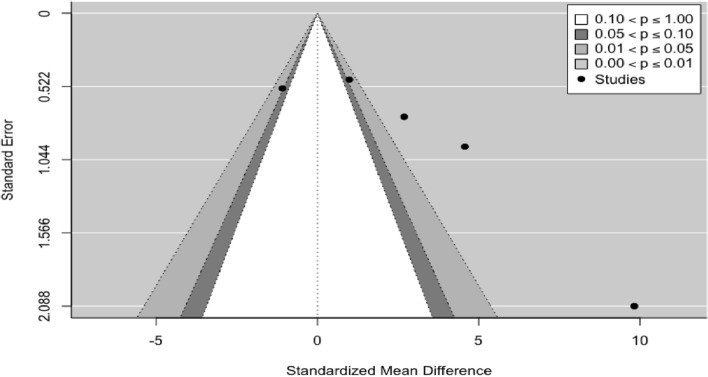
Funnel plot for the regeneration in new cementum

Considering the fit to a random-effects model, the combined variance, ${\tau }^{2}$ was 6.1573 with a percentage of total heterogeneity ${I}^{2}$ between studies of 92.33% (Fig. 2). At a significance level of 0.05 there was no statistical evidence to confirm that the effect between treatments is equal. There was high variability between the results obtained when comparing both treatments *Q* = 52.1251 (*p* < 0.0001), meaning therefore that there was great influence by at least one experimentation (stem cells). The Funnel plot, the influence graph and the forest plot show the significant differences between the experiments, differences that are attributed to the study by Zhou et al. 2012 [40] that has a share of the study of 13.63%, and an estimated average effect size at a confidence level of 0.95 (95%) of between 5.74 and 13.92 (Fig. 3).

#### New bone

According to the results obtained when comparing the regeneration of the alveolar bone, the effect size between Group 1 and Group 2, with Group 1 being stem cells and Group 2 other regenerative materials, it was evident that Group 1 presented greater regeneration of periodontal tissue than Group 2. Indeed, the results showed that among the reviewed studies the effect size had an average of 1.2717 mm of tissue, with a standard deviation of 0.3806. This therefore means that the average effect size explicitly expresses the highest value by Group 1 compared to Group 2, with characteristics of experimental homogeneity in each of the experiments resulting from a coefficient of variation, not greater than one, | cv |< 1.

**Fig. 4:**
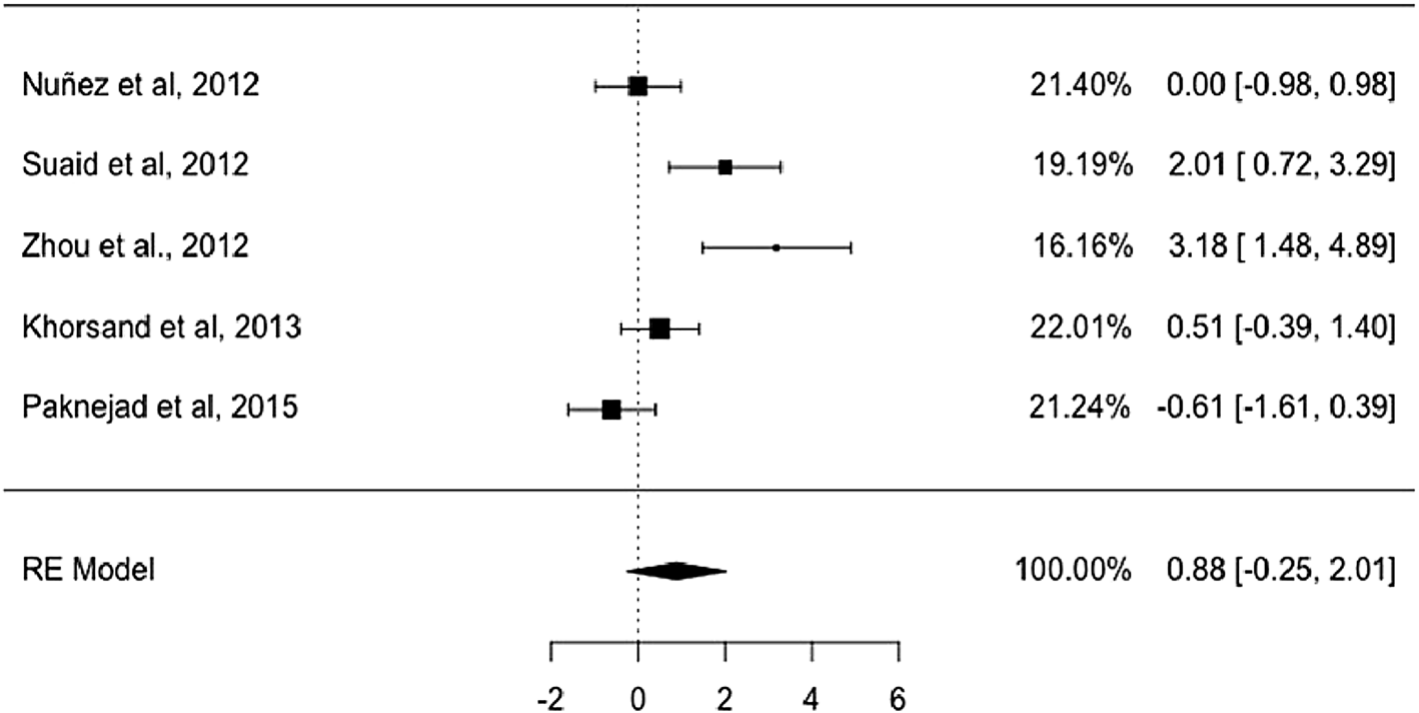
Forest plot and data from the meta-analysis for the regeneration in alveolar bone. ${\tau }^{2}$ (estimated amount of total heterogeneity): 1.3108 (SE = 1.2021). $\tau$ (square root of estimated ${\tau }^{2}$ value): 1.1449. ${I}^{2}$ (total heterogeneity / total variability): 80.46%. *H*^2^ (total variability/sampling variability) : 5.12%. Test for Heterogeneity: *Q* (df = 4) = 20.4717, *p*-value: 0.0004

**Fig. 5:**
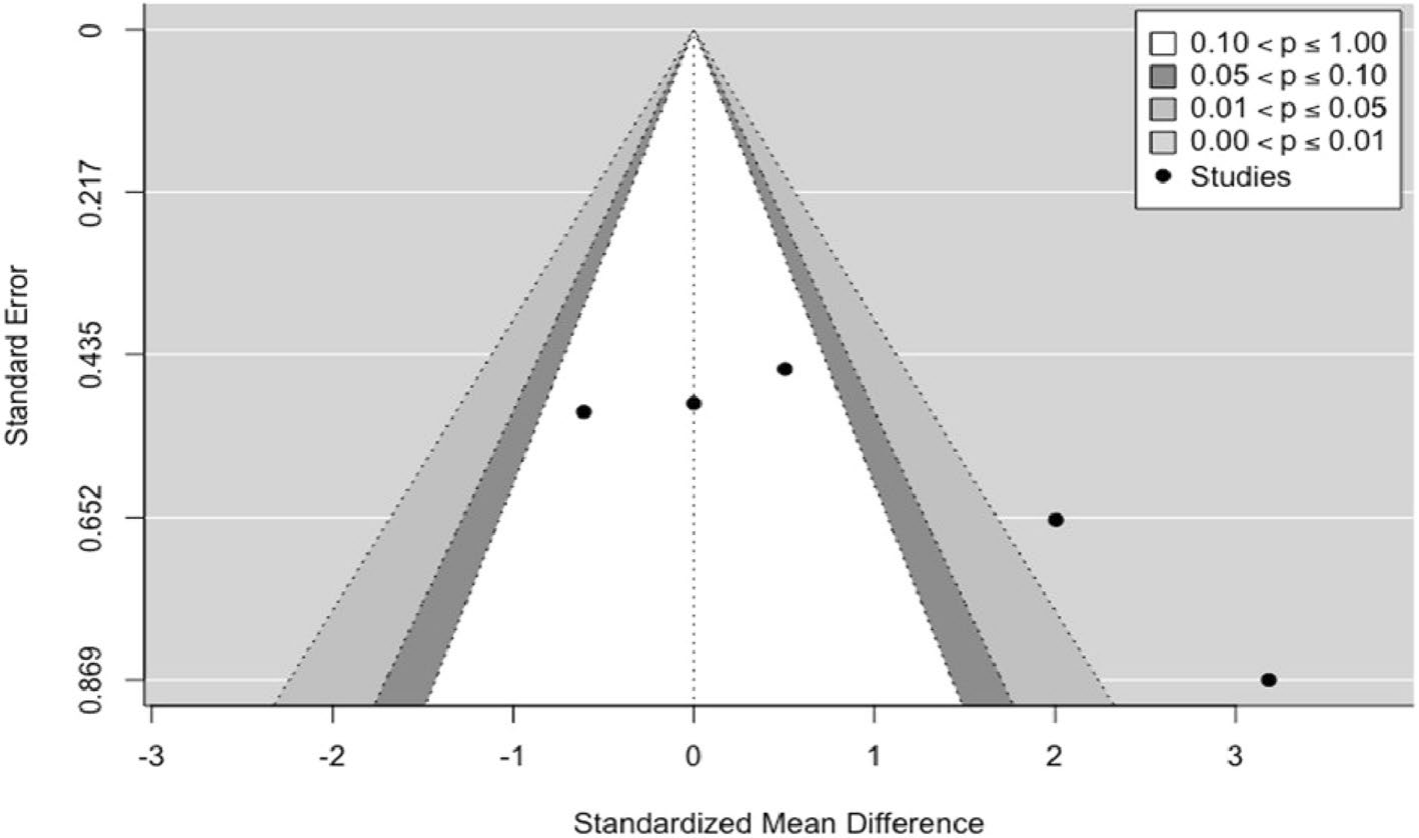
Funnel plot for the regeneration in alveolar bone

After calculating the effect size in each of the 5 studies, a high variability was determined in each of the experiments (Group with stem cells and group with other materials), an average of 1.2717 ± 0.3806 mm with characteristics of experimental homogeneity in each of the experiments due to a coefficient of variation not greater than one, | cv |< 1. Considering the fit to a random effects model, the combined variance ${\tau }^{2}$ was 1.3108, with a percentage of total heterogeneity ${I}^{2}$ between studies of 80.46% (Fig. 4). At a significance level of 0.05 there was no statistical evidence to suggest that the effect between treatments is equal. Therefore, there was a high variability between the results obtained when comparing both treatments. *Q* = 20.4717 (*p* < 0.0004). The Funnel plot, the influence plot and the forest plot show that there were significant differences between the experiments, and these differences were attributed to the study by Zhou et al*.* 2012 [40] that has a share of the study of 16.16% and an estimated average effect size at a confidence level of 0.95 (95%) of between 1.48 and 4.89 (Fig. 5).

#### New periodontal ligament

It was not possible to develop the meta-analysis due to the lack of data.

## Discussion

The present systematic review and meta-analysis observed that periodontal regeneration with MSCs alone or mixed with other regenerative materials, such as beta-tricalcium phosphate, bovine bone or platelet-rich plasma, offered better regenerative results than those attained for the group with only regenerative materials. Qualitative studies showed that PDLSc and BMSCs appear to have greater regenerative properties. After reviewing the literature, two systematic review articles on periodontal regeneration with MSCs published by Tassi et al*.* [49] and Yan et al*.* [50] were found. In the first study, the meta-analysis was not possible because of the heterogeneities observed in the study designs. In the second study, the meta-analysis showed no statistically significant differences in effect between PDLSCs and BMScs.

An ideal bone graft substitute must have certain properties, which include osteoconduction, osteoinduction, osteoincorporation, osteointegration, and osteogenesis [51]. Despite several efforts to invent and characterize various bone graft substitutes, none of these could be accepted as an ideal alternative to autografts due to the low ability of the bone substitutes to enhance osteoinduction and osteogenesis [52, 53]. The majority of in vitro and many in vivo studies have suggested that the MSCs have the potential to increase osteoinduction and osteogenesis [46], in particular in association with bone substitute materials [41]. The use of β-TCP is applicable as a scaffold for BMSC transplantation and it helps to augment alveolar bone without affecting cementum regeneration [46].

The regenerative potential of MSCs is probably related predominantly to the stage of differentiation and lineage commitment of the cells, as well as proliferation rates, heterogeneity of selectively isolated MSCs subpopulations, the number of cells transferred to the defects and the scaffold composition and three-dimensional arrangement [54, 55].The origin of stem cells and the role these play in the regenerative processes has been the subject of much debate, sometimes with contradictory results. Nagahara et al*.* [46] demonstrated that periodontal regeneration with BMSCs with beta-tricalcium phosphate was enhanced at 8 weeks in alveolar cementum and alveolar bone. Nevertheless, Iwasaki et al*.* [44] determined that there was a considerable significant difference in periodontal regeneration in PDLSCs with beta-tricalcium phosphate and collagen compared to the BMSCs.

Bone regeneration by gene transfer into MSCs has also been reported; however, the reported transduction efficiency into MSCs by each vector was not always high. Chung et al. [31] indicated that when using MSCs with adenovirus BMP-2 (advBMP-2) in bone defects, the periodontal regeneration was significantly better at 8 weeks than in the control group. Other studies have indicated that PDLSCs, have the same results [32, 34, 39] without needing to use viral vectors. Fawzy El-Sayed et al*.* [33] used gingival margin stem progenitor cells (GMSCs) together with IL-1ra-releasing hyaluronic acid synthetic extracellular matrix (HA-sECM), and they concluded that there was a significant periodontal regenerative potential compared to the control groups. On the other hand, in the case of platelet-rich plasma with MSCs and autologous bone, there was no significant difference between the platelet-rich plasma alone and the autologous bone group [38]. Chondrogenic differentiation of MSCs before implantation is also a useful strategy for the regeneration of the cartilage; however, its role in alveolar bone and periodontal ligament regeneration is still not clear [45].

The main limitations of this research were related to the design of the experiments (different and non-equitable groups), the different types of animal model (dog, pig, and rat), the high variability of MSCs, and the different methodology used to apply the cells, whether alone or in combination with many different types of biomaterials.

In the present study, there were significant differences in the use of MSCs compared to the group of other biomaterials for periodontal regeneration. The most commonly used stem cells were periodontal ligament and bone marrow stem cells, and these cells were mixed with other regenerative biomaterials, obtaining better results in periodontal regeneration. Taking into account the results attained from the meta-analyses, it is possible to conclude that stem cells have a higher periodontal regenerative capacity than other single regenerative materials.
